# Broader phenotypic traits and widespread brain hypometabolism in spinocerebellar ataxia 27

**DOI:** 10.1111/joim.13052

**Published:** 2020-03-19

**Authors:** M. Paucar, J. Lundin, T. Alshammari, Å. Bergendal, M. Lindefeldt, M. Alshammari, G. Solders, J. Di Re, I. Savitcheva, T. Granberg, F. Laezza, E. Iwarsson, P. Svenningsson

**Affiliations:** 1 Departments of Clinical Neuroscience, Karolinska Institutet; 2 Neurology, Karolinska University Hospital; 3 Molecular Medicine and Surgery, Karolinska Institutet, Stockholm, Sweden; 4 The University of Texas Medical Branch, Galveston, TX, USA; 5 Pharmacology and Toxicology, College of Pharmacy, King Saud University, Riyadh, Saudi Arabia; 6 Pediatric Neurology, Astrid Lindgren’s Hospital; 7 Neurophysiology, Karolinska University Hospital, Stockholm, Sweden; 8 Neuroscience Graduate Program, The University of Texas Medical Branch, Galveston, TX, USA; 9 Departments of, Nuclear Medicine; 10 Radiology, Karolinska University Hospital, Stockholm, Sweden

**Keywords:** spinocerebellar ataxia type 27, *FGF14*, positron emission tomography, intellectual disability, psychosis

## Abstract

Paucar M, Lundin J, Alshammari T, Bergendal Å, Lindefeldt M, Alshammari M, Solders G, Di Re J, Savitcheva I, Granberg T, Laezza F, Iwarsson E, Svenningsson P (Karolinska Institutet; Karolinska University Hospital; Astrid Lindgren’s Hospital, Stockholm, Sweden; The University of Texas Medical Branch, Galveston, TX, USA; King Saud University, Riyadh, Saudi Arabia). Broader phenotypic traits and widespread brain hypometabolism in spinocerebellar ataxia 27.

**Objective.:**

The goal of this study was to characterize a Swedish family with members affected by spinocerebellar ataxia 27 (SCA27), a rare autosomal dominant disease caused by mutations in fibroblast growth factor 14 (*FGF14*). Despite normal structural neuroimaging, psychiatric manifestations and intellectual disability are part of the SCA27 phenotype raising the need for functional neuroimaging. Here, we used clinical assessments, structural and functional neuroimaging to characterize these new SCA27 patients. Since one patient presents with a psychotic disorder, an exploratory study of markers of schizophrenia associated with GABAergic neurotransmission was performed in *fgf14*^−/−^ mice, a preclinical model that replicates motor and learning deficits of SCA27.

**Methods.:**

A comprehensive characterization that included clinical assessments, cognitive tests, structural neuroimaging studies, brain metabolism with ^18^F-fluorodeoxyglucose PET ([18F] FDG PET) and genetic analyses was performed. Brains of *fgf14*^−/−^ mice were studied with immunohistochemistry.

**Results.:**

Nine patients had ataxia, and all affected patients harboured an interstitial deletion of chromosome 13q33.1 encompassing the entire *FGF14* and integrin subunit beta like 1 (*ITGBL1*) genes. New features for SCA27 were identified: congenital onset, psychosis, attention deficit hyperactivity disorder and widespread hypometabolism that affected the medial prefrontal cortex (mPFC) in all patients. Hypometabolism in the PFC was far more pronounced in a SCA27 patient with psychosis. Reduced expression of VGAT was found in the mPFC of *fgf14*^−/−^ mice.

**Conclusions.:**

This is the second largest SCA27 family identified to date. We provide new clinical and preclinical evidence for a significant psychiatric component in SCA27, strengthening the hypothesis of *FGF14* as an important modulator of psychiatric disease.

## Graphical Abstract

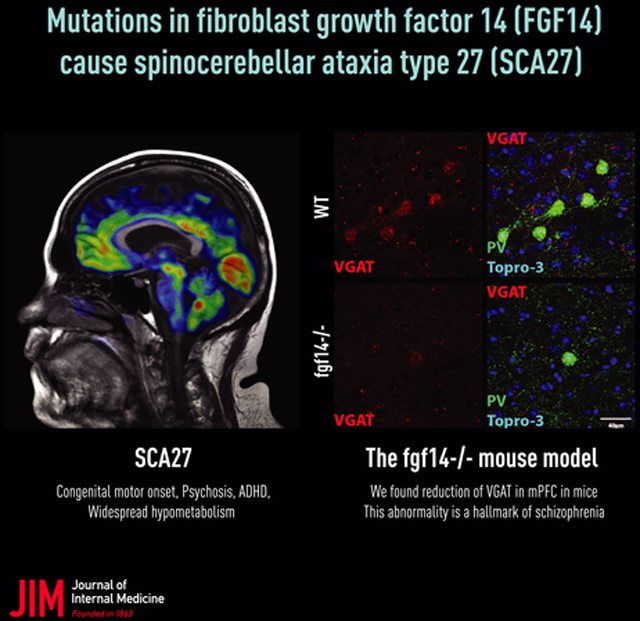

## Introduction

Spinocerebellar ataxias (SCA) are a heterogeneous and growing group of autosomal dominant disorders. Whilst the majority (~60%) of SCA cases can be attributed to pathological CAG repeat expansions in different genes, others have been linked to rare single-gene mutations. Because of the complexity of clinical presentation and significant phenotype overlap, diagnosis of SCA on pure clinical grounds is challenging, requiring complementary genetic analyses, especially for the rare SCA subtypes such as spinocerebellar ataxia type 27 (SCA27). SCA27 features slowly progressive cerebellar ataxia, variable psychiatric and behavioural symptoms, and intellectual disability (ID) associated in some cases with axonal polyneuropathy. The first description of SCA27 in a large Dutch family reported a missense mutation (F145S) in fibroblast growth factor 14 (*FGF14*) [1], a gene also reported in the literature as fibroblast homologous factor 4 or FHF4. Only seven families [1–7] and seven single patients with *FGF14* mutations have been reported [8–14], whereas screening in three ataxia cohorts identified one case only [2, 15, 16]. Only two of nine patients in the Dutch SCA27 family had cerebellar atrophy [1] suggesting that alterations in brain connectivity are more prominent than structural changes and therefore raising the need for functional imaging. Age of onset (AO) and the clinical presentation of SCA27 are variable; other phenotypes than SCA27 associated with mutations in *FGF14* include episodic ataxia [5, 6, 12, 14], paroxysmal chorea [8], parkinsonism [7] and dysmorphism and/or microcephaly [3, 9, 10]. Despite a growing literature, no clear genotype–phenotype correlations have been established yet. Experimental evidence for the original *FGF14*^*F145S*^ mutation reported in the Dutch family supports a dominant negative mechanism [17].

Here, we describe a Swedish family extending over four generations with nine patients affected with SCA27 caused by an interstitial ~600 kb deletion in chromosome 13q33.1. This deletion affects the entire coding regions of the *FGF14* and integrin subunit beta like 1 (*ITGBL1*) genes. This family displays new features for SCA27 such as congenital onset, psychosis with self-harming behaviour, attention-deficit/hyperactivity disorder (ADHD) and widespread brain hypometabolism. In addition, reduced expression of VGAT, a presynaptic component of inhibitory synapses and a marker of schizophrenia, was found in the medial prefrontal cortex (mPFC) of *fgf14*^−/−^ mice, an animal model of SCA27 which replicates motor and cognitive features of the disease. Our studies provide new clinical and preclinical evidence for a wider spectrum of psychiatric features in SCA27.

## Patients and methods

The study was approved by the local ethical board in Stockholm; informed consent was obtained from all the participants and/or from a guardian. Patients underwent a comprehensive assessment that included physical examination, psychometric evaluations, neurophysiological tests and neuroimaging with MRI and brain ^18^F-fluorodeoxyglucose PET [18F] FDG PET (Supplementary Material).

### Animals

The primary *fgf14*^−/−^ colony was maintained at the animal facilities of the University of Texas Medical Branch following approved protocols. Genotypes were confirmed by either in-house PCR analysis or Charles River Laboratories International, Inc. (Houston, TX, USA). Description of the animal husbandry is provided in the Supplementary Material.

### Immunofluorescence

Brain tissue derived from *fgf14*^−/−^ and *fgf14*^+/+^ 4- to 5-month-old male (*n* = 1 per group) and female (*n* = 2 per group) mice was processed for immunofluorescence staining using either 4% paraformaldehyde followed by permeabilization, blocking, and primary and Alexa-conjugated secondary antibody staining as previously described [18] and outlined in Supplementary Material.

### Data acquisition and image analysis

Multi-channel confocal images were acquired using a Zeiss LSM-510 META confocal microscope as previously described [18]. Detailed information on image quantification can be found in Supplementary Material.

## Results

**Fig. 1:**
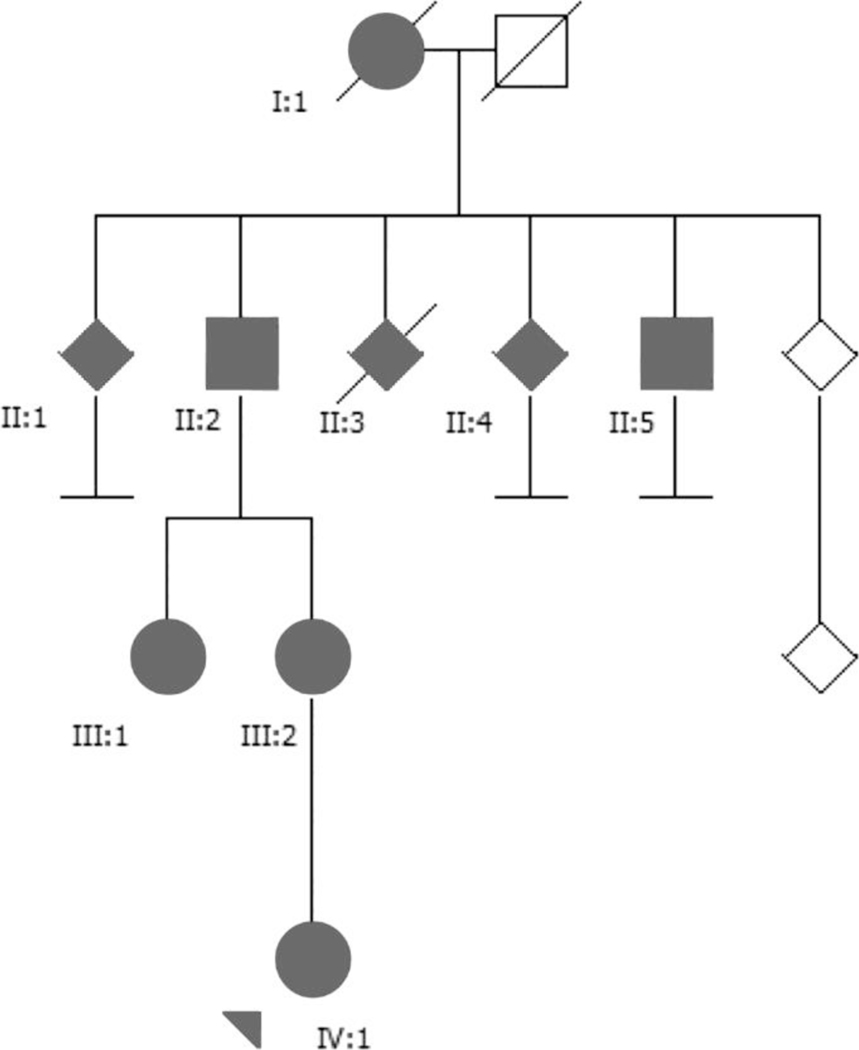
Pedigree for a Swedish family with several patients affected by SCA27 caused by the deletion (600 kb) of chromosome 13q33.1 encompassing the entire FGF14 and ITGBL1 genes. This is the largest deletion associated with SCA27 reported so far. Patients II:1, II:3 and II:4 were assigned as symptomatic based on history and patient I:1 by review of charts. Five patients (II:2, II:5, III:1, III:2 and IV:1) were evaluated for this characterization. [Correction added on 20 April 2020, after first online publication: In Figure 1, the pedigree was shown in red colour in the online version and has been converted to black in this current version.]

Nine patients were affected, patients II:1, II:3 and II:4 were assigned as symptomatic based on history provided by relatives and another (I:1) by review of charts (Fig. 1). Five patients were interviewed and examined by movement disorder neurologists. Patient II:2 consented to participate, but declined imaging studies and cognitive assessments.

### Phenotype

Briefly, all affected patients but the index case (IV:1) displayed predominant axial ataxia, and AO was variable. Appendicular ataxia was more predominant in the index case. Cognitive profiles ranged from low (II:5, IV:1), borderline level (III:2) to manifest ID (III:1); notably, all the examined patients have attended special schools or have had extra support during school (III:2 and IV:1) (Table 1). Patient IV:1 never learned to read or write. Dysmetria, gaze-evoked nystagmus and impaired optokinetic nystagmus were also present (Table 2; Supplementary Material). None of the studied patients had dysmorphism, paroxysmal chorea, episodic ataxia or pyramidal abnormalities. Reflexes were normal in all examined patients except for III;1 who had hyporeflexia and polyneuropathy. The index case (patient IV:1) had anger outbursts, and she and her mother were both diagnosed with ADHD. Treatment attempt with metylphenidate exacerbated her anger outburst, whilst a subsequent treatment with dexamphetamine induced tachycardia, motivating interruption. Patient III:1 has type 1 diabetes mellitus (T1DM). At age 19, she presented with delusions, hearing and occasionally visual hallucinations and self-harming behaviour, leading to diagnosis of psychosis and emotionally unstable personality disorder. She has been treated with neuroleptics ever since. At age 25, she presented with progressive gait and balance difficulties, action tremor and slurred speech. Besides marked axial ataxia and gross tremor, extrapyramidal signs (EPS) were evident. Patient III:2 had congenital cervical dystonia, tremor and nystagmus initially exacerbated by fever. Her dystonia resolved spontaneously, but the time point of remission was undetermined; four patients had minipolymyoclonus (II:2, II:5; III:2 and IV:1).

### Neuroimaging

All the examined patients but the index case had variable cerebellar atrophy (more predominant in the vermis), and patient III:2 had atrophy of the cervical spinal cord. Patients II:1, II:5 and III:1 had variable degree of cortical atrophy, which was progressive in patient II:5.

**Fig. 2:**
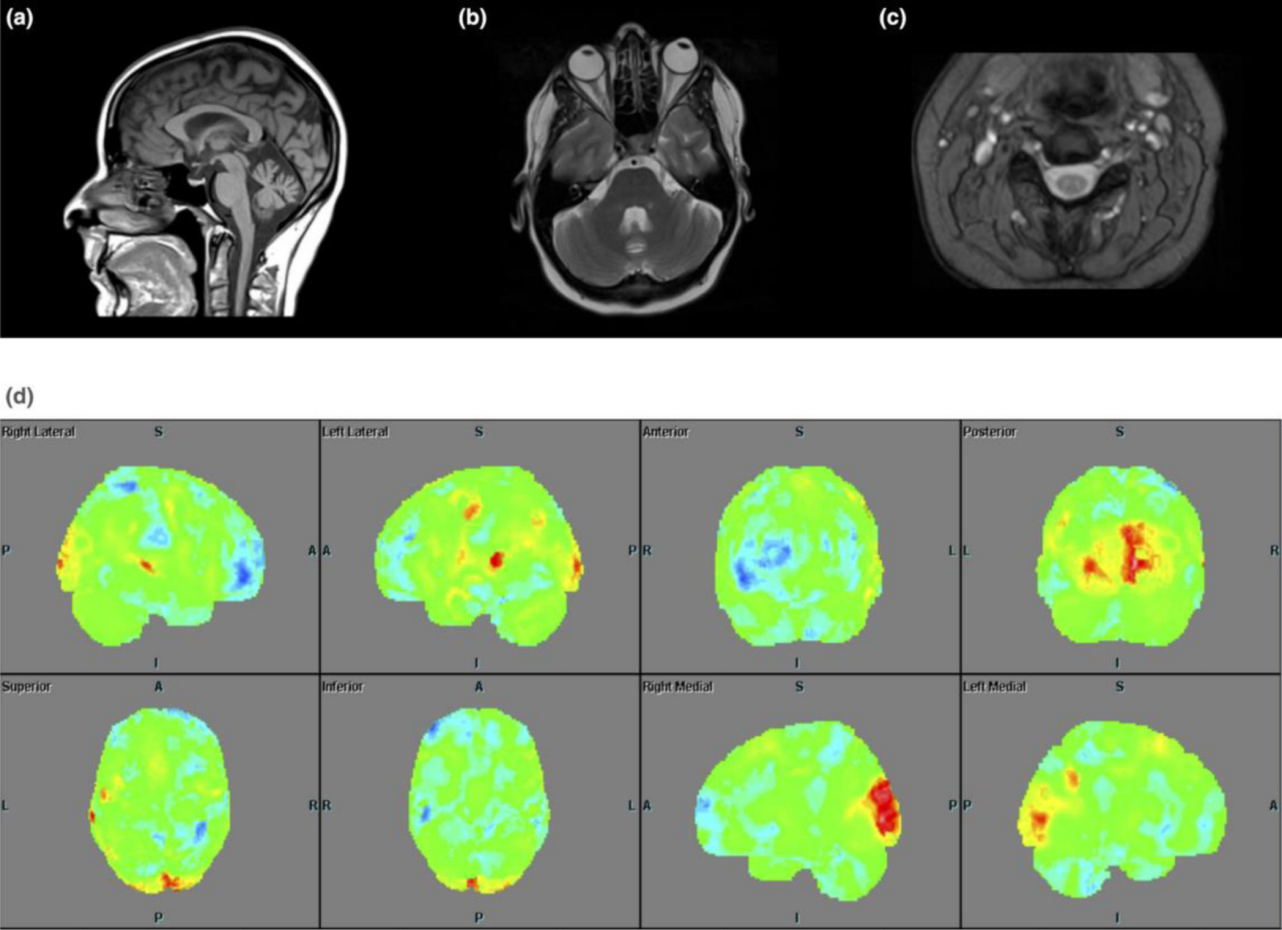
Brain and cervical MRI in patient III:2 at 34 years of age on a 1.5 T scanner showing mild to moderate vermal atrophy (a: mid-sagittal T1-weighted imaging, b: 4 mm thick slice; axial T2-weighted imaging, 5 mm thick slice) and mild atrophy of cervical spinal cord (c: T2*-weighted imaging at the C3-level, 4 mm thick slice). (d) Widespread moderate hypometabolism is evident on this 3D-SSP figure; the patient was not on any psychotropic medication. The affected areas are PFC, temporal cortex, putamen, vermis and pons. No shown is cingulate gyrus.

**Fig. 3:**
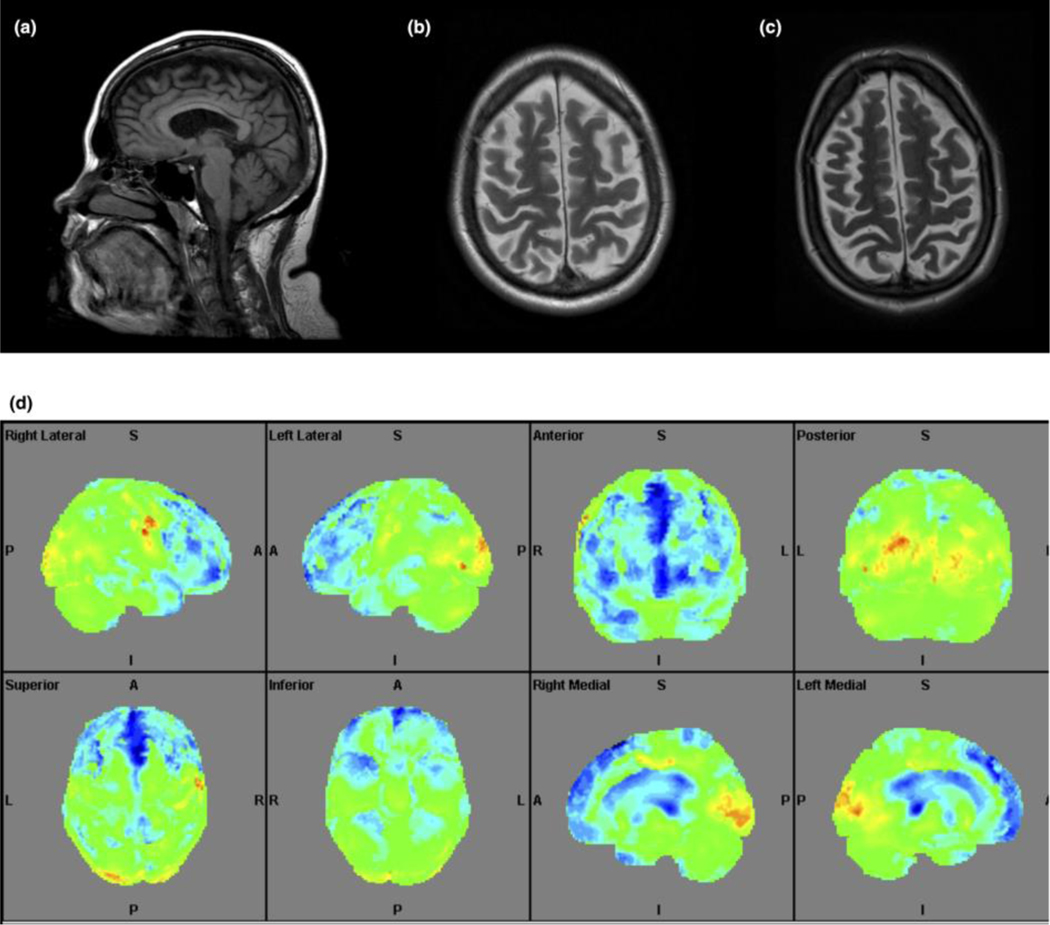
Brain MRI in Patient III:1 at 43 and 47 years of age (a) and 47 years of age (b and c). (a) Subtle atrophy of the vermis (sagittal T1-weighted imaging, 4 mm thick slices). (b) Moderate but progressive cortical atrophy (axial T2-weighted imaging, 4 mm thick slices; images not aligned similarly making the difference hard to appreciate on one slice). (d) 3D-SSP figure displaying marked hypometabolism in the PFC, part of temporal lobe and anterior cerebellum. Cingulate gyrus and thalamus are no shown.

**Fig. 4:**
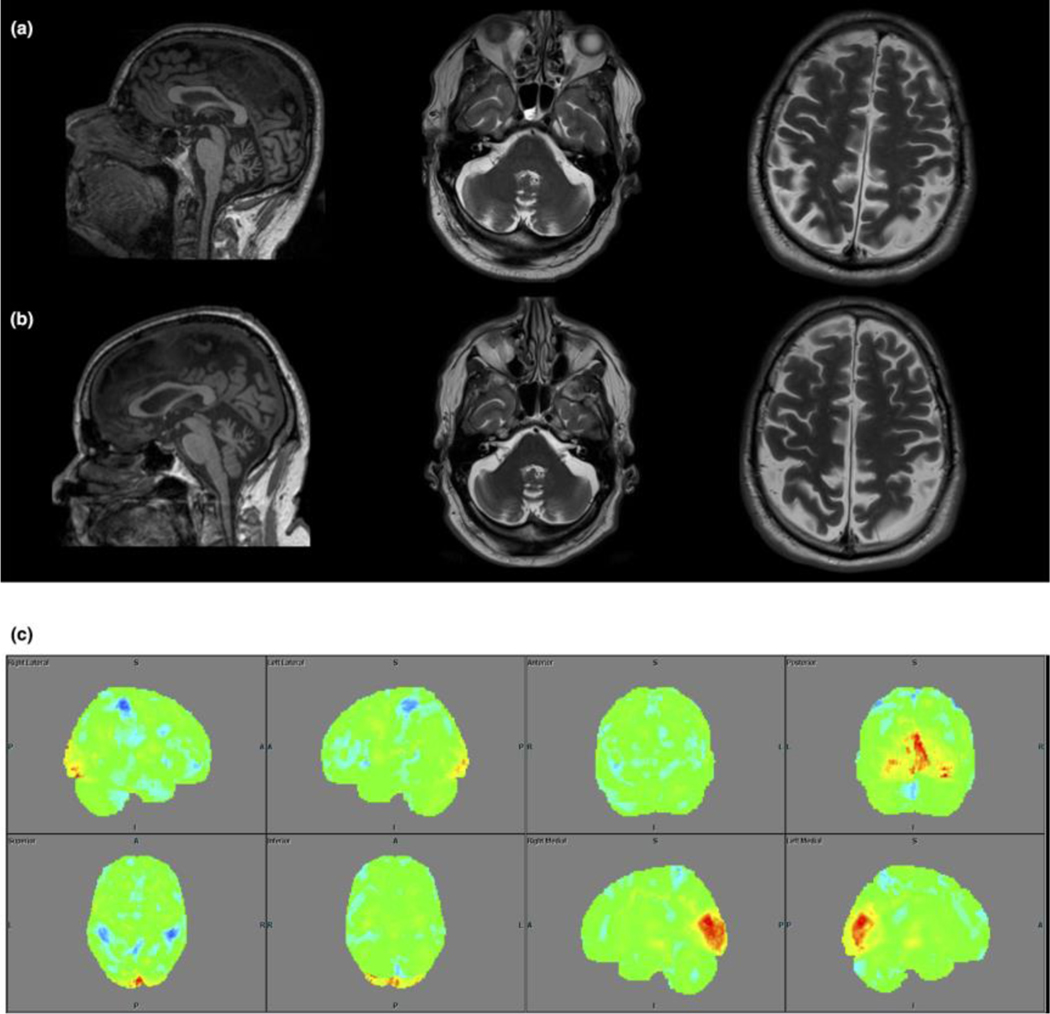
Brain MRI on the same 3T scanner in patient II:5 at 60 (left column) and 64 (right column) years of age displaying moderate cortical and infratentorial atrophy on axial T2-weighted imaging (first two rows, 4/3 mm thick slices, respectively) and mid-sagittal T1-weighted imaging (last row, 1/1.2 mm thick slices, respectively). Figure 3b 3D-SSP for FDG PET in the same patient displaying scattered areas with hypometabolism.

All examined patients had hypometabolism in the prefrontal cortex (PFC), temporal cortex and cerebellum (Summarized in Table 3 and shown as Figs 2, 3 and 4). This hypometabolism was particularly severe and widespread in patient III:1 who also had hypermetabolism in the putamina. Patient III:1 also presented with hypometabolism in the cingulate gyrus, a pattern that was present also in patient III:2, albeit less pronounced.

### Genotype

**Fig. 5:**
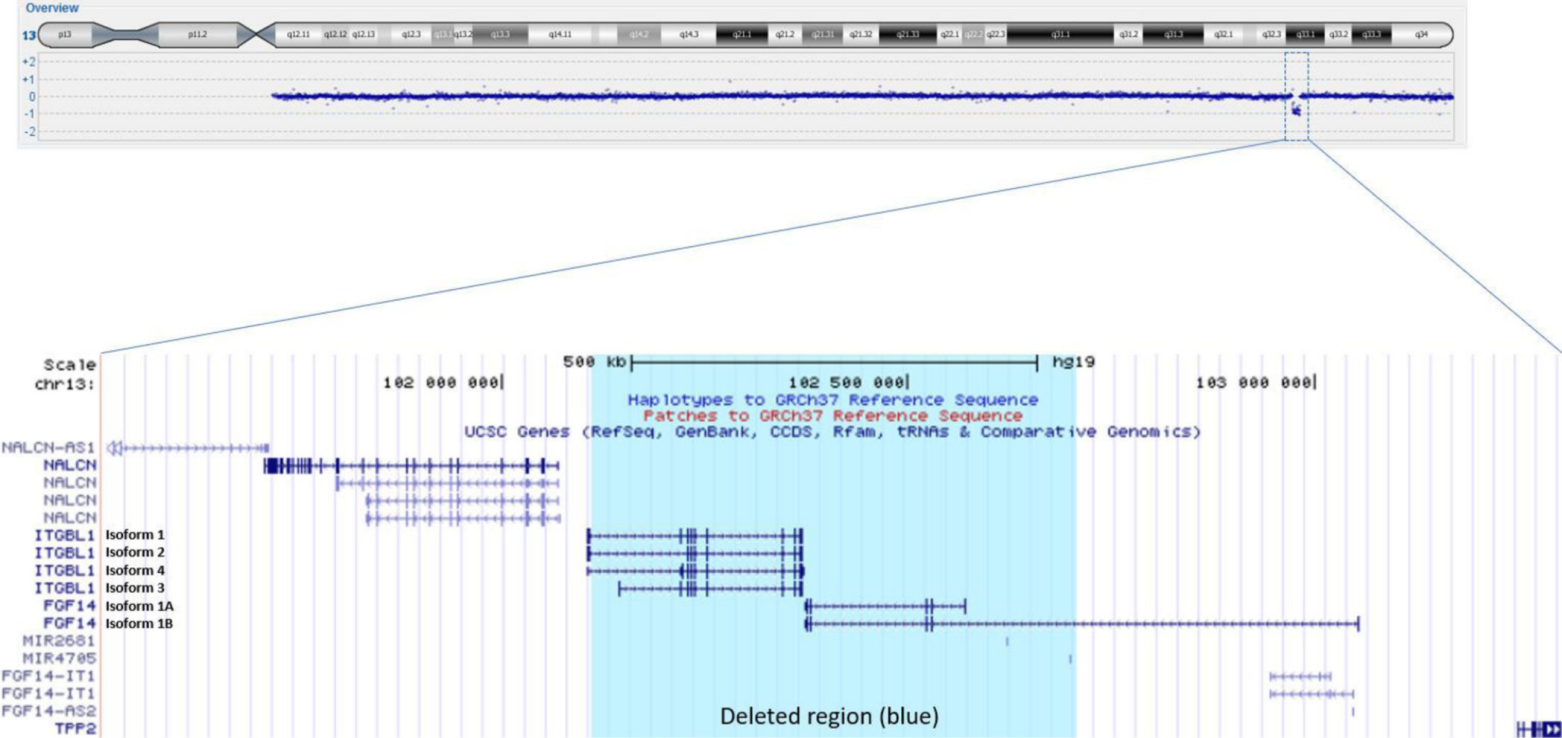
Ideogram of chromosome 13 showing the deletion on 13q33.1 detected from the array-CGH analysis. The deleted region (chr13: 102 108 769–102 707 510 according to Hg19) is highlighted in blue and encompasses the FGF14 and ITGBL1 genes.

Pathological nucleotide expansions for SCA1, 2, 3, 6, 7, 8, 12 and dentatorubropallidoluysian atrophy (DRPLA) were ruled out in the index case. Array comparative genomic hybridization (aCGH) revealed that this patient as carrier of an interstitial 599kb deletion on chromosome 13q33 that segregated with the disease (found in patients II:2, II:5, III:1 and III:2). The deletion affects the entire coding regions of *FGF14* and *ITGBL1* (Fig. 5). The minimum deleted region (hg19) is chromosome 13: 102 108 769–102 707 510 includes the entire (exon 1–5) FGF14 transcript 1 and part of (exon 2–5) of FGF14 transcript 2 as well as the whole ITGBL1 transcript variant 3, exon 3–11 of transcript 1 and exon 2–10 of transcript 2 and exon 2– 11 of transcript 4 of the *ITGBL1* gene.

### Prefrontal cortex in fgf14^−/−^ mice

**Fig. 6:**
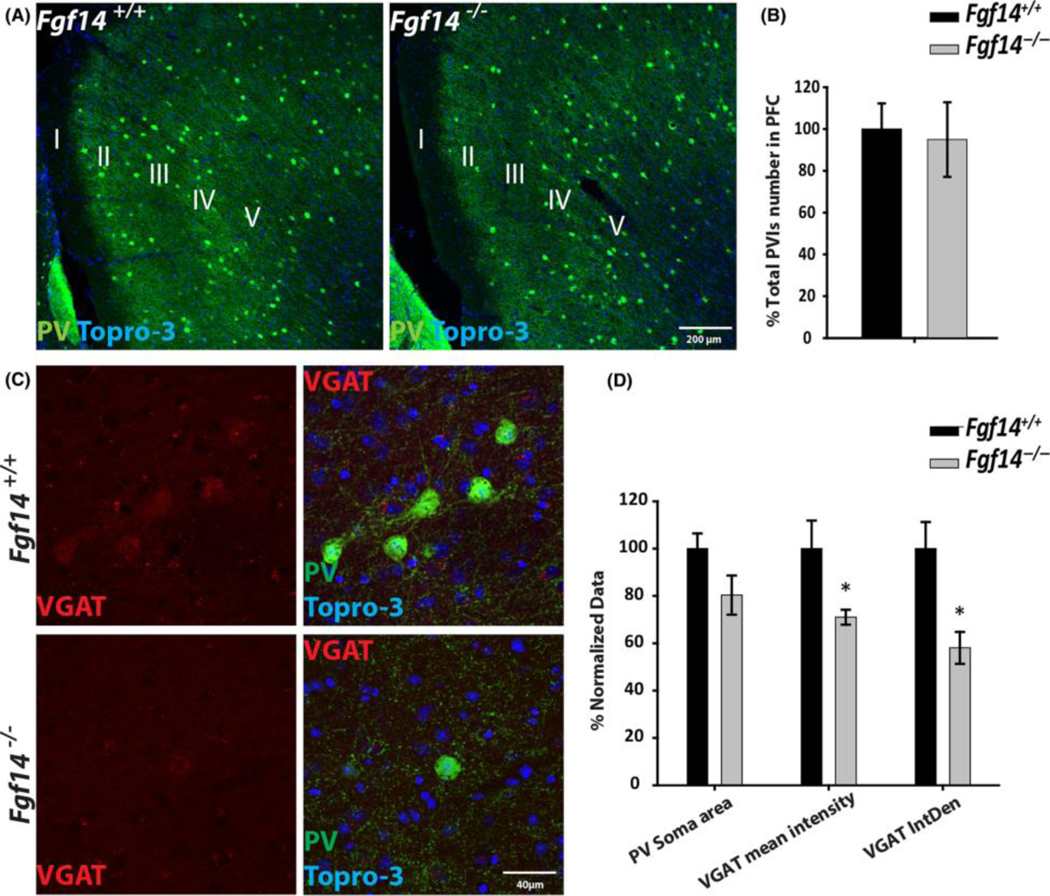
VGAT expression in PV interneurons in PFC. (a) Representative confocal images of PV interneurons immunofluorescence (green), the blue channel represents Topro-3 nuclei staining, in the PFC of fgf14^+/+^ and fgf14^−/−^ mice. (b) Quantification of total PV interneurons in PFC visualized by Topro-3 nuclei staining in indicated genotypes. (c) VGAT expression in PV interneurons in PFC layer III, merged image of green channel representing PV, red representing VGAT, and blue channel represents Topro-3 nuclei staining in indicated genotypes. (d) Plot represents quantification of PV soma area, VGAT mean fluorescence intensity and VGAT integrated fluorescence intensity measured in PV interneuron somas in indicated genotypes. Data represent mean SEM, (n = 3 mice/genotype, one section/mouse), and statistical differences were assessed by nonparametric Mann–Whitney test; *P < 0.05.

We used immunohistochemistry and confocal imaging in a small group of animals to stain the mPFC for parvalbumin (PV) positive interneurons and one of the GABAergic inhibitory markers, the vesicular GABAergic transporter (VGAT) (Fig. 6). PV interneurons are a major source of inhibitory synaptic terminals in cortical circuits, and loss of GABAergic signalling originating from PV interneurons is a common feature in neuropsychiatric disorders associated with cognitive impairment. Amongst molecules involved in regulating GABA, VGAT is considered a biomarker of schizophrenia as it is downregulated in postmortem tissue from patients. Image quantification based on immunofluorescence staining revealed that the total number of PV-positive cells in the mPFC of this cohort of animals was not significantly reduced in *fgf14*^−/−^ mice compared to *fgf14*^+*/*+^ controls (Fig. 6a,b, 125 15 in wild type controls versus 118 22, *P* = 0.82, in *fgf14*^−/−^ mice, *n* = 3 per group). Similarly, the area of the PV-positive cells soma was not significantly different in *fgf14*^−/−^ versus *fgf14*^+*/*+^ mice (Fig. 6c,d). However, the expression level of VGAT in the soma of PV-positive cells, which represents the primary source of the enzyme cytoplasmic pool, was significantly reduced in *fgf14*^−/−^ mice compared to *fgf14*^+*/*+^ control (71% ± 3, 100% ± 11 *P* < 0.05, and 58% ± 6, 100% ± 11, *P* < 0.05, respectively, *n* = 3 per group, Fig. 6c,d). These results strengthen the importance of FGF14 in regulating signalling associated with schizophrenia and other psychiatric disorders and support clinical data of psychiatric traits in SCA27 patients described here.

## Discussion

This is the second largest SCA27 family described to date and the first time a complete heterozygous deletion of the *FGF14* gene is reported. Complete penetrance with variable expressivity, slow progression rate and motor exacerbation is similar to previous descriptions of SCA27 cases [1–4, 13, 18]. Nevertheless, congenital onset, cervical dystonia and widespread brain hypometabolism constitute new traits for SCA27; early AO has been described before but only in a handful SCA27 cases [3, 4, 6, 8– 10]. Only the index case in our family had anger outbursts whereas depressive symptoms affected some members of the first reported SCA27 family [1, 19]. Psychosis, self-harming behaviour and ADHD constitute other new clinical observations. Similar to patients with SCA27, structural anomalies in the cerebellum or basal ganglia are not found in the *fgf14*^−/−^ mouse model despite the presence of motor and memory deficits [20, 21]. In addition, lambs affected by episodic ataxia harbouring the mutation c.46C> T in *FGF14* lack also gross histopathological abnormalities [22].

EPS in patient III:1 are likely a side effect of treatment with neuroleptics and her polyneuropathy a possible complication to T1DM; however, polyneuropathy occurs in some SCA27 patients [1– 3]. Thirteen mutations and one variant of unclear significance (VUS) in *FGF14* have been described; four of them are 13q33.1 deletions of variable size [4, 6, 9, 10]. A microdeletion (95kb) was associated with impaired gait, delayed motor and language development, low IQ and mild dysmorphism, whereas a larger deletion (202 kb), affecting partially both *FGF14* and *ITGBL1*, was associated with ataxia exacerbated by fever, but with normal IQ [4, 9]. *ITGBL1* is expressed in the cerebral cortex, cerebellum, retina and spinal cord, but is function for neuronal function is still unknown. In our family, *ITGBL1* was also deleted and one patient had motor exacerbation triggered by fever. However, whether the somewhat larger deletion of *ITGBL1* contributes to phenotype severity in our family is unclear. A larger *FGF14* deletion (441 kb) was associated with a severe phenotype that included ataxia, delayed motor milestones, microcephaly, moderate ID, marked dysmorphism and progressive cerebellar atrophy [10]. The fourth deletion (424 kb) was associated with ID and episodic ataxia that slowly progressed into permanent ataxia [6]. In addition, two SCA27 cases were caused by translocations t(5;13) and t(13;21), both disrupting the *FGF14* gene and associated with variable ID. One of these cases featured ataxia, microcephaly, severe ID and polyneuropathy, whereas the second patient had paroxysmal chorea and mild ID [3, 8]. Small deletions are associated with either normal IQ or mild ID [4, 9], but neither microcephaly nor dysmorphism has been described in conventional *FGF14* mutations. In summary, moderate to marked ID is present in all patients with larger *FGF14* deletions [6, 10] in contrast to the apparent milder ID reported for conventional mutations [1, 2]. Our results seem to strengthen the association between *FGF14* deletion and ID, but missing data on cognitive performance of SCA27 patients from previous publications preclude further comparisons.

Structural imaging in SCA27 is normal in most cases (78%); cerebellar atrophy has been described only in four patients, two of them with long disease duration or in association with microcephaly [1, 10, 13]. However, this figure may be underestimated due to a lack of longitudinal neuroimaging studies. One SCA27 patient with facial dyskinesias from the original Dutch family had reduced striatal dopamine transporter binding upon SPECT examination but FDG PET was not performed [19]. Brain hypometabolism correlated with lower IQ, showing patterns affecting the PFC, temporal regions and the vermis in all patients. Deficits in the PFC are in line with a frontal-executive dysfunction and similar to the channelopathy SCA19/22 [23]. Besides those areas, thalamus and the anterior part of the cingulate gyrus are hypometabolic in untreated schizophrenia patients [24]; striatal hypermetabolism may reflect long-standing medication with neuroleptics [25]. Hypometabolism in the frontal regions characterizes un-medicated ADHD patients [26]. The widespread brain hypometabolism in patient III:1 has to be interpreted with caution though due to long-standing medication with psychotropic drugs.

Anatomical and functional abnormalities in the dorsolateral PFC are considered hallmarks of cognitive impairment in schizophrenia and other psychiatric disorders [27]. In these complex brain pathologies, cognitive impairment is thought to derive from unbalance between the excitatory and inhibitory tone in hippocampus and PFC [28, 29]. Evidence indicates that reduced connectivity between PV interneurons and excitatory cells in the hippocampus and PFC could be the initial cause of circuitry dysfunction leading to decreased gamma frequency oscillations and functional uncoupling between the two brain regions, resulting in cognitive impairment [30–32]. Signs of disrupted GABAergic transmission have been found consistently across both postmortem brains and experimental models that recapitulate cognitive deficits in schizophrenia [30, 33]. Furthermore, therapies aiming at normalizing the inhibitory tone in the brain have shown pro-cognitive effects in preclinical studies and clinical trials for the treatment of schizophrenia-associated cognitive defects indicating that the GABAergic system is both a marker of the disease as well as therapeutic target [34].

Previous studies conducted in male *fgf14*^−/−^ mice found a significant decrease in markers of GABAergic transmission in the hippocampus compared to wildtype controls, a phenotype that was accompanied by reduction in inhibitory postsynaptic currents, *in vivo* gamma frequency and working memory [18]. Additionally, in the same study bioinformatics analysis of schizophrenia transcriptomics revealed functional co-clustering of FGF14 and genes enriched within the GABAergic pathway along with correlatively decreased expression of FGF14 and VGAT in the disease context. Disruption of FGF14 in the *fgf14*^−/−^ experimental model also leads to paroxysmal dyskinesia and ataxic gait attributed to dysfunction of GABA signalling in the basal ganglia that are remarkably similar to the motor features of SCA27 and paroxysmal hyperkinesias in some *FGF14* mutations [20]. Thus, the *fgf14*^−/−^ mouse model is a valid experimental model that recapitulates a broad spectrum of molecular, cellular motor and nonmotor features, such as cognitive and behavioural abnormalities of patients with SCA27.

Here, we used the *fgf14*^−/−^ mouse model to corroborate the anatomical and functional changes in the PFC observed in this newly identified SCA27 family. We focused on one marker of GABAergic transmission, VGAT, which is the vesicular GABAergic transporter found consistently downregulated in schizophrenic patients [35] and in experimental models associated with the disease [18, 36]. Using immunohistochemistry, we confirmed a significant decrease in VGAT in the PFC of *fgf14*^−/−^ mice, confirming a key role of FGF14 in brain areas associated with the cognitive domain in the context of psychiatric and neurological disorders [37].

Expression of *FGF14* in the murine central nervous system starts during embryogenesis and continues into adulthood [20], and FGF14 acts intracellularly by binding to voltage-gated Na^+^ (Nav) channels promoting their location to the axon initial segment [38–40]. FGF14 has also been shown to regulate presynaptic Ca_v_2 Ca^2+^ channels, voltage-gated K^+^ channels and presynaptic neurotransmitter recycling in granule cells in the cerebellum [19]. In addition, FGF14 is necessary for synaptic plasticity in the hippocampus and is required for neurogenesis in the dentate gyrus [18, 36, 41]. Alterations in those ion channels in *fgf14*^−/−^ mice provide the molecular basis for the striking clinical resemblance with ataxia channelopathies [5]. As complementary evidence of FGF14’s role in human hereditary ataxias, several studies indicate a role of FGF14 in cerebellar function in rodent models. At the cellular level, FGF14 has been found expressed in both granule neurons and Purkinje neurons where it forms a complex at the axon initial segment with the Nav channels and contributes to repetitive firing by modulation of Na^+^ transient and persistent currents [42, 43]. At the behavioural level, *fgf14*^−/−^ mice exhibit severe motor deficits with ataxic gait, consistent with a role of FGF14 in the cerebellum [20]. These phenotypes are however accompanied by paroxysmal dyskinesias [20], learning and memory deficits [21], and a variety of other behavioural deficits such as aggressivity and disturbances in sexual behaviour [44], supporting the much broader role of FGF14 in the brain as indicated by the heterogeneous presentation of clinical symptoms in SCA27 patients.

Taken together, we have identified the second largest SCA27 family adding to the knowledge on haploinsufficiency for this disease. In addition, we report a wide range of novel clinical features and widespread frontal hypometabolism. Moreover, our findings are in accordance with the fact that polymorphisms in *FGF14* have emerged as significant risk factors not only for schizophrenia but also for other psychiatric conditions [37, 45]. Future neuropathological studies will provide more insights on SCA27 at the regional and cellular levels.

## Supplementary Material

Supplementary material

## Figures and Tables

**Table 1:** Summary of cognitive features in two patients from the SCA 27 family

Cognitive domain	Neuropsychological tests	Patient II:5 (at age 63 years), z-score	Patient III:1 (at age 47 years), z-score

Brief cognitive status examination	MoCA (Montreal cognitive assessment)	−4.27*	−6.1*
	MMT (Mini-Mental Test)	−2.63*	NA
General intellectual ability	Ravens progressive matrices IQ	−0.5, IQ = 92	−2.33*, IQ = 65
Verbal episodic memory	RAVLT (Rey Auditory Verbal Learning Test) learning	−0.88	−3.32*
	RAVLT retention	−0.34	−2.74*
Visuo-spatial episodic memory	ROCFT (Rey Osterrieth Complex Figure Test) immediate recall	−3*	<−3*
	ROCFT delayed recall	−3*	NA
Working memory	Digit span/WAIS-IV	−2*	−2*
Spatial/visual construction	ROCFT copy	−7.99*	−6.22*
Information processing speed	SDMT (Symbol Digit Modalities Test)	−3.45*	−5.11*
	FAS/COWAT (Controlled Oral Word Association Test)	NA	−1.45*
Motor speed	FT (Finger-tapping test) dominant hand	−1.25	NA		
	FT (nondominant hand)	−0.81	NA		

* Significant cognitive deficits defined as a *z*-score of —1.5 standard deviations below the norm (marked with). The index case (IV:1) was evaluated on two occasions at a different centre, and her IQ scale ranged between 72 and 82. IQ scale for Patient III:2 was 71.

NA, not assessed.

**Table 2:** *Clinical features found in a Swedish SCA27 family harbouring a* ~*600 kb deletion on chromosome 13q33 affecting both* FGF14 *and* ITGBL-1

Patient	Age of motor onset (years)/Current age or age of death	Symptom/s at motor onset	SARA at first/last examination (age in years)	INAS at last examination	ENeG	Functional stage at last examination (FARS)	Eye movements	Psychiatric symptoms	Cognitive assessment	Comorbidities

I:1	23/87	Tremor	NA/NA (83)	NA	NA	5	Nystagmus	NA	NA	NA
II:2	30/70	Gait disorder	18 (66)/NA^a^	2	NA	4	Nystagmus^b^	Normal	MoCA = 22 p	Thrombophilia^c^, spinal stenosis, multiple falls and fractures
II:5	18/64	Gait disorder	17 (60)/18.5 (63)	3	Carpal tunnel syndrome	4	Nystagmus	Normal	Illiteracy MoCA = 18 p IQ scale = 92	Rheumatoid arthritis
III:1	25/48	Gait disorder and tremor	17 (43)/19.5 (48)	3^d^	Sensori-motor PNP	4	Nystagmus	Emotionally unstable personality disorder, Psychotic syndrome Depression^e^	Intellectual disability MoCA = 19 p IQ scale = 65	Type 1 diabetes mellitus, Obesity (BMI = 35.4)
III:2	Neonatal/39	Cervical dystonia and tremor exacerbated by fever	5.5 (33)/7 (39)	1	Normal	1	Nystagmus	ADHD, Dyslexia	Motor and language delay MoCA = 26 p IQ scale = 71	Overweight (BMI = 28.6)
IV:1	6/18	Postural tremor	4 (13)/5 (18)	2	Normal	2	Nystagmus	Anger outbursts, ADHD (Diagnosed at age 8)	Moderate ID IQ scale = 72–82	Hypothyroidism (From age 4), Fever seizures, Obesity (BMI = 30)

Functional stage (0–6) according to the Friedreich ataxia rating scale (FARS).

INAS, Inventory of nonataxia signs; MoCA, Montreal cognitive assessment; NA, Not assessed; PNP, Polyneuropathy; SARA, Scale for the assessment and rating of ataxia.

a The patient progressed after this visit requiring a walker.

b Nystagmus was present in all directions.

c Due to heterozygous mutation in factor 2.

d Extrapyramidal signs were found (mild posturing, bradykinesia and slowness), likely due to treatment with neuroleptics. All patients had optokinetic nystagmus, and minipolymyoclonus was evident in patients II:2, II:5; III:2 and IV:1.

e These prominent psychiatric features were present long before motor onset.

**Table 3:** Neuroimaging results in five patients with SCA27 harbouring a deletion if chromosome 13q33.1

Patient	Structural neuroimaging modality (age at exam in years)	Findings on structural neuroimaging	Pattern of FDG PET uptake

I:1	CT (81)	Mild cortical atrophy (GCA 0–1) A few WMA (Fazekas 1)	NA
II:5	Brain and cervical MRIs (60, 63, 64)	Moderate cortical (GCA 1–2) and cerebellar atrophy (vermis and superior cerebellar hemispheres). Mild central atrophy with slow progression over time No WMA A few microbleeds in both deep and lobar regions	Mild hypometabolism in PFC, temporal cortex, cerebellum and vermis
III:1	Brain CTs (8 examinations: 34–42) Brain MRI (43, 47) Cervical spine MRI (43)	Moderate cortical (GCA 1–2) and mild central atrophy with slow progression. Subtle atrophy of the vermis and cerebellum No WMA	Severe hypometabolism in PFC, temporal cortex, cingulate gyrus, parts of cerebellum and thalami Relative hypermetabolism in putamina.
III:2	Brain, cervical and upper thoracic MRI (34)	Mild cortical atrophy (GCA 0–1) and mild-moderate vermal and cervical spine atrophy No WMA	Moderate hypometablism in ventromedial parts of the PFC, temporal cortex, cingulate gyrus, part of right parietal cortex, putamina, parts of cerebellum and pons
IV:1	Brain MRI (11) Brain CT (17)	Normal findings: No atrophy No WMA A 0, Fazekas 0	NA

GCA, global cortical atrophy rating scale; NA, not available; PFC, Prefrontal cortex; WMA, white matter abnormalities.

## References

[1] van Swieten JC, Brusse E, de Graaf BM A mutation in the fibroblast growth factor 14 gene is associated with autosomal dominant cerebellar ataxia. Am J Hum Genet 2003; 72: 191–9. 12489043 10.1086/345488 PMC378625

[2] Dalski A, Atici J, Kreuz FR Mutation analysis in the fibroblast growth factor 14 gene: frameshift mutation and polymorphisms in patients with inherited ataxias. Eur J Hum Genet 2005; 13: 118–20. 15470364 10.1038/sj.ejhg.5201286

[3] Misceo D, Fannemel M, Barøy T SCA27 caused by a chromosome translocation: further delineation of the phenotype. Neurogenetics 2009; 10: 371–4. 19471976 10.1007/s10048-009-0197-x

[4] Coebergh JA, Fransen van de Putte DE, Snoeck IN A new variable phenotype in spinocerebellar ataxia 27 (SCA 27) caused by a deletion in the FGF14 gene. Eur J Paediatr Neurol 2014; 18: 413–5. 24252256 10.1016/j.ejpn.2013.10.006

[5] Choquet K, La Piana R, Brais B. A novel frameshift mutation in FGF14 causes an autosomal dominant episodic ataxia. Neurogenetics 2015; 16: 233–6. 25566820 10.1007/s10048-014-0436-7

[6] Amado A, Blanco MO, Repáraz-Andrade A. Spinocerebellar Ataxia 27: Clinical Phenotype of Twin Sisters with FGF14 Deletion. Neuropediatrics 2017; 48: 131. 28192817 10.1055/s-0037-1598110

[7] Groth CL, Berman BD. Spinocerebellar Ataxia 27: A review and characterization of an evolving phenotype. Tremor Other Hyperkinet Mov 2018; 30: 1–6. 10.7916/D80S0ZJQ PMC5801325 29416937

[8] Shimojima K, Okumura A, Natsume J Spinocerebellar ataxias type 27 derived from a disruption of the fibroblast growth factor 14 gene with mimicking phenotype of paroxysmal non-kinesigenic dyskinesia. Brain Dev. 2012; 34: 230–3. 21600715 10.1016/j.braindev.2011.04.014

[9] Tucker M, Kalb F, Escobar L. Infant spinocerebellar ataxia type 27: early presentation due to a 13q33.1 microdeletion involving the FGF14 gene. J Genet Syndr Gene Ther 2013; 4: 4–11.

[10] Planes M, Rooryck C, Vuillaume ML SCA27 is a cause of early-onset ataxia and developmental delay. Eur J Paediatr Neurol 2015; 19: 271–3. 25530029 10.1016/j.ejpn.2014.11.013

[11] Hadjivassiliou M, Martindale J, Shanmugarajah P Causes of progressive cerebellar ataxia: prospective evaluation of 1500 patients. J Neurol Neurosurg Psychiatry 2017; 88: 301–9. 27965395 10.1136/jnnp-2016-314863

[12] Choi KD, Kim JS, Kim HJ Genetic variants associated with episodic ataxia in Korea. Sci Rep 2017; 7: 1–11. 29062094 10.1038/s41598-017-14254-7 PMC5653837

[13] Miura S, Kosaka K, Fujioka R Spinocerebellar ataxia 27 with a novel nonsense variant (Lys177X) in FGF14. Eur J Med Genet 2019; 62: 172–6. 30017992 10.1016/j.ejmg.2018.07.005

[14] Schesny M, Joncourt F, Tarnutzer AA. Acetazolamide-responsive episodic ataxia linked to novel splice site variant in FGF14 gene. Cerebellum 2019; 18: 649–53. 30607796 10.1007/s12311-018-0997-3

[15] Stevanin G, Durr A, Dussert C Mutations in the FGF14 gene are not a major cause of spinocerebellar ataxia in Caucasians. Neurology 2004; 63: 936–7. 15365159 10.1212/01.wnl.0000137020.30604.1e

[16] Chen Z, Li X, Tang B Spinocerebellar ataxia type 27 (SCA27) is an uncommon cause of dominant ataxia among Chinese Han population. Neurosci Lett 2012; 520: 16–19. 22579694 10.1016/j.neulet.2012.05.008

[17] Laezza F, Gerber BR, Lou JY The FGF14(F145S) mutation disrupts the interaction of FGF14 with voltage-gated Na+ channels and impairs neuronal excitability. J Neurosci 2007; 27: 12033–44. 17978045 10.1523/JNEUROSCI.2282-07.2007 PMC6673376

[18] Alshammari TK, Alshammari MA, Nenov MN Genetic deletion of fibroblast growth factor 14 recapitulates phenotypic alterations underlying cognitive impairment associated with schizophrenia. Transl Psychiatry 2016; 6: e806. 27163207 10.1038/tp.2016.66 PMC5070049

[19] Brusse E, de Koning I, Maat-Kievit A Spinocerebellar ataxia associated with a mutation in the fibroblast growth factor 14 gene (SCA27): a new phenotype. Mov Disord 2006; 21: 396–401. 16211615 10.1002/mds.20708

[20] Wang Q, Bardgett ME, Wong M Ataxia and paroxysmal dyskinesia in mice lacking axonally transported FGF14. Neuron 2002; 35: 25–38. 12123606 10.1016/s0896-6273(02)00744-4

[21] Wozniak DF, Xiao M, Xu L Impaired spatial learning and defective theta burst induced LTP in mice lacking fibroblast growth factor 14. Neurobiol Dis 2007; 26: 14–26. 17236779 10.1016/j.nbd.2006.11.014 PMC2267915

[22] Dittmer KE, Jolly RD, Mayhew IG Familial episodic ataxia in lambs is potentially associated with a mutation in the fibroblast growth factor 14 (FGF14) gene. PLoS One 2017; 12: e0190030. 10.1371/journal.pone.0190030 PMC5734737 29253853

[23] Paucar M, Bergendal Å, Gustavsson P Novel features and abnormal pattern of cerebral glucose metabolism in spinocerebellar ataxia 19. Cerebellum 2018; 17: 465–76. 29527639 10.1007/s12311-018-0927-4 PMC6028832

[24] Hazlett EA, Vaccaro DH, Haznedar MM F-18Fluorodeoxyglucose positron emission tomography studies of the schizophrenia spectrum: The legacy of Monte S. Buchsbaum, M.D. Psychiatry Res 2019; 271: 535–40. 30553101 10.1016/j.psychres.2018.12.030

[25] Szechtman H, Nahmias C, Garnett ES Effect of neuroleptics on altered cerebral glucose metabolism in schizophrenia. Arch Gen Psychiatry 1988; 45: 523–32. 2897836 10.1001/archpsyc.1988.01800300019002

[26] Lou HC, Henriksen L, Bruhn P. Focal cerebral hypoperfusion in children with dysphasia and/or attention deficit disorder. Arch Neurol 1984; 41: 825–9. 6331818 10.1001/archneur.1984.04050190031010

[27] Zierhut KC, Schulte-Kemna A, Kaufmann J Distinct structural alterations independently contributing to working memory deficits and symptomatology in paranoid schizophrenia. Cortex 2013; 49: 1063–72. 23040316 10.1016/j.cortex.2012.08.027

[28] Gao R, Penzes P. Common mechanisms of excitatory and inhibitory imbalance in schizophrenia and autism spectrum disorders. Curr Mol Med 2015; 15: 146–67. 25732149 10.2174/1566524015666150303003028 PMC4721588

[29] Sawada K, Young CE, Barr AM Altered immunoreactivity of complexin protein in prefrontal cortex in severe mental illness. Mol Psychiatry 2002; 7: 484–92. 12082566 10.1038/sj.mp.4000978

[30] Ferguson BR, Gao WJ. PV Interneurons: critical regulators of E/I balance for prefrontal cortex-dependent behavior and psychiatric disorders. Front Neural Circuits 2018; 12: 1–13. 29867371 10.3389/fncir.2018.00037 PMC5964203

[31] Perez SM, Boley A, Lodge DJ. Region specific knockdown of Parvalbumin or Somatostatin produces neuronal and behavioral deficits consistent with those observed in schizophrenia. Transl Psychiatry 2019; 9: 1–13. 31636253 10.1038/s41398-019-0603-6 PMC6803626

[32] Woo TU, Spencer K, McCarley RW. Gamma oscillation deficits and the onset and early progression of schizophrenia. Harv Rev Psychiatry 2010; 18: 173–89. 20415633 10.3109/10673221003747609 PMC2860612

[33] Taylor SF, Tso IF. GABA abnormalities in schizophrenia: a methodological review of in vivo studies. Schizophr Res 2015; 167: 84–90. 25458856 10.1016/j.schres.2014.10.011 PMC4409914

[34] Xu MY, Wong AHC. GABAergic inhibitory neurons as therapeutic targets for cognitive impairment in schizophrenia. Acta Pharmacol Sin 2018; 39: 733–53. 29565038 10.1038/aps.2017.172 PMC5943898

[35] Hoftman GD, Volk DW, Bazmi HH Altered cortical expression of GABA-related genes in schizophrenia: illness progression vs developmental disturbance. Schizophr Bull 2015; 41: 180–91. 24361861 10.1093/schbul/sbt178 PMC4266281

[36] Wearne TA, Parker LM, Franklin JL, Goodchild AK, Cornish JL. GABAergic mRNA expression is differentially expressed across the prelimbic and orbitofrontal cortices of rats sensitized to methamphetamine: relevance to psychosis. Neuropharmacology 2016; 111: 107–18. 27580848 10.1016/j.neuropharm.2016.08.038

[37] Di Re J, Wadsworth PA, Laezza F. Intracellular fibroblast growth factor 14: emerging risk factor for brain disorders. Front Cell Neurosci 2017; 11: 1–7. 28469558 10.3389/fncel.2017.00103 PMC5396478

[38] Hsu WC, Scala F, Nenov MN CK2 activity is required for the interaction of FGF14 with voltage-gated sodium channels and neuronal excitability. FASEB J 2016; 30: 2171–86. 26917740 10.1096/fj.201500161 PMC4871802

[39] Shavkunov AS, Wildburger NC, Nenov MN The fibroblast growth factor 14·voltage-gated sodium channel complex is a new target of glycogen synthase kinase 3 (GSK3). J Biol Chem 2013; 288: 19370–85. 23640885 10.1074/jbc.M112.445924 PMC3707642

[40] Ali SR, Liu Z, Nenov MN Functional modulation of voltage-gated sodium channels by a FGF14-based peptidomimetic. ACS Chem Neurosci 2018; 9: 976–87. 29359916 10.1021/acschemneuro.7b00399 PMC6033619

[41] Alshammari MA, Alshammari TK, Nenov MN Fibroblast growth factor 14 modulates the neurogenesis of granule neurons in the adult dentate gyrus. Mol Neurobiol 2016; 53: 7254–70. 26687232 10.1007/s12035-015-9568-5 PMC4916041

[42] Bosch MK, Carrasquillo Y, Ransdell JL Intracellular FGF14 (iFGF14) is required for spontaneous and evoked firing in cerebellar purkinje neurons and for motor coordination and balance. J Neurosci 2015; 35: 6752–69. 25926453 10.1523/JNEUROSCI.2663-14.2015 PMC4412895

[43] Shakkottai VG, Xiao M, Xu L FGF14 regulates the intrinsic excitability of cerebellar Purkinje neurons. Neurobiol Dis 2009; 33: 81–8. 18930825 10.1016/j.nbd.2008.09.019 PMC2652849

[44] Hoxha E, Marcinnò A, Montarolo F Emerging roles of Fgf14 in behavioral control. Behav Brain Res 2019; 356: 257–65. 30189289 10.1016/j.bbr.2018.08.034 PMC10082543

[45] Sowers ML, Re JD, Wadsworth PA Sex-specific proteomic changes induced by genetic deletion of fibroblast growth factor 14 (FGF14), a regulator of neuronal ion channels. Proteomes 2019;7: 1–17. 10.3390/proteomes7010005 PMC6473632 30678040

