# Supplementary material for: Broader phenotypic traits and widespread brain hypometabolism in spinocerebellar ataxia 27
Source: J Intern Med. Author manuscript; Available in PMC 2023 Apr 24. (PMC10123866; doi:10.1111/joim.13052)
Supplement: Supplementary material [file NIHMS1883460-supplement-Supplementary_material.docx]

**Supplementary material**

**Broader phenotypic traits and widespread brain hypometabolism in spinocerebellar ataxia 27**

Martin Paucar; Johanna Lundin; Tahani Alshammari; Åsa Bergendal; Marie Lindefeldt, Musaad Alshammari; Göran Solders; Jessica Di Re; Irina Savitcheva; Tobias Granberg; Fernanda Laezza; Erik Iwarsson; and Per Svenningsson.

1. **Methods:**

Clinical assessments: Patients went through a comprehensive characterization that included physical examination with Scale for the Assessment and Rating of Ataxia (SARA) and inventory of non-ataxia symptoms (INAS); Montreal Cognitive Assessment (MoCA), genotyping, nerve conduction studies with electroneurography (ENeG), electromyography (EMG) and neuroimaging with magnetic resonance imaging (MRI) and ^18^F-fluorodeoxyglucose positron emission tomography ([^18^F] FDG-PET). Psychometric assessments for Patients II:5 and III:1 were administered by the same investigator using standardized batteries as previously described^1^. The index case went through psychometric evaluations at a different setting, Patient III:2 was evaluated by a different investigator. For Patient I:1 medical records and neuroimaging studies from a different hospital were reviewed. For the other patients, we went through earlier medical records and evaluated the patients for a course of 5 to 6 years.

**1.1 Structural neuroimaging**

Clinical 3 Tesla MRI scanners were used for this characterization. Volumetric assessment in Patient II:5 was performed using FreeSurfer^2^ and it was also intended for the other patients but they either declined or did not comply with scheduled exams. In Patient III:1, motion artifacts precluded volumetric analysis. White matter abnormalities (WMA) was assessed with Fazekas’ rating scale and cortical atrophy was evaluated according to the global cortical atrophy (GCA) rating scale^3,4^. FDG-PET studies were performed on the same scanner at the Karolinska University Hospital in Huddinge according to previous descriptions^1^.

1**.2 FDG-PET**

Standardized comparisons with healthy individuals were performed using three-dimensional stereotactic surface projection (3D-SSP) software and automated volume of interest-based analysis of FDG uptake^1^.

1. **Animal studies**

**2.1 Animal husbandry**

*Fgf14*^−/−^ and *fgf14*^+/+^ male and female mice are maintained on an inbred C57/BL6J background with greater than ten generations of backcrossing to C57/BL6J. Animals were bred in the UTMB animal care facility: either heterozygous *fgf14^+/−^* males and females or, in few cases, homozygotes *fgf14*^−/−^ males with *fgf14^+/−^* females; *fgf14*^+/+^ wild-type mice served as control. Both male and female mice were used in this study at 4–6 months of age, unless otherwise stated. The University of Texas Medical Branch operates in compliance with the United States Department of Agriculture Animal Welfare Act, the Guide for the Care and Use of Laboratory Animals, and IACUC approved protocols. Mice were housed, n≤5 per cage, kept under a 12-h light/12-h dark cycle with sterile food and water ad libitum.

**2.2 Immunofluorescence of brain sections**

Preparation and staining of mouse brain sections were performed as previously described^5^. In brief, the mice were deeply anesthetized with 2,2,2-tribromoethanol (250 mg/kg i.p.; Sigma-Aldrich, Saint Louis, MO) diluted in 1X PBS (Toe pinch will be applied before proceeding with the procedure to ensure the animal is deeply anesthetised and then briefly perfused intracardially (flow rate: 8-10 ml/min for 2-5 min) with 1X PBS. Followed by 10 min of 4% paraformaldehyde freshly prepared (Sigma-Aldrich) in 1X PBS); all solutions were adjusted to pH 7.4. To ensure complete tissue fixation, brains were removed carefully and post-fixed into the same fixative for 1h at 4°C and then cryopreserved in 20-30% sucrose/PBS at 4°C in preparation for sectioning. Brains were then embedded in OCT compound (Tissue-Tek®, Ted Pella, Inc) and sectioned sagittally into 20–25 µm slices using a Leica CM1850 cryostat (Leica Microsystems, Buffalo Grove, IL) and slices stored in a cryoprotectant solution (ethylene glycol based; 30% ethylene glycol, 30% glycerol, 10% 0.2M sodium phosphate buffer pH 7.4, in dH2O) at −20°C. Free floating sections were washed with 1X PBS, then pre-incubated with a permeabilizing agent (1% Triton X-100, 0.5% Tween 20 in 1X PBS) for 10 min followed by 30 min 1X PBS washing. Sections were then incubated with a blocking buffer (10% normal goat serum NGS (Sigma-Aldrich) in 1X TBS containing 0.3% Triton X-100 for 1 hr. Finally, sections were incubated overnight at 4°C on an orbital rotator with primary antibodies in 3% bovine serum albumins BSA (Sigma-Aldrich) in 1X PBS containing 0.1% Tween 20. Primary antibodies used in this study were: rabbit anti-parvalbumin (1:1000, Abcam catalog number Ab11427); and mouse anti-VGAT (1:500, Novus Biological, catalog number CL2793). Following the overnight primary antibody incubation, sections were washed five times with 1X PBS or TBS buffer solution, incubated with the appropriate Alexa secondary antibodies at a 1:250 dilution in 3% BSA/PBST, then washed five more times with buffer solution. Prior to mounting on Superfrost® glass microscope slides (Fisher Scientific, Waltham, MA) with ProLong® Gold anti-fade, slices were rinsed with water and counter **stained using the nuclear marker Topro-3 (1–3000, Life Technologies).**

**2.3 Confocal microscopy**

Confocal images were acquired using the Zeiss LSM-510 META confocal microscope with a Plan-NeoFluar (10×/0.3) objective and Plan-Apochromat (63×/1.46 Oil) objective, with consistent gain and offset settings, as well as a number of confocal image Z-stacks across experimental sets. Multitrack acquisition was performed with excitation lines at 488 nm for Alexa 488, 543 nm for Alexa 568, and 633 nm for A647. Z-series stack confocal images were taken at fixed intervals: 0.4 µm for 63× with the same pinhole setting for all three channels; frame size was 1024 × 1024.

**2.4 Data acquisition and image analysis**

Images were analyzed using ImageJ US NIH (http://imagej.nih.gov/ij). For total number of PV interneuronses and soma fluorescence intensity analysis, Z-stacks of confocal images were sum-projected, an ROI corresponding to soma was highlighted using an intensity threshold method, and total cell number, mean fluorescence intensity and integrated intensity were quantified.

1. **Results**
   1. **Clinical findings**

Briefly, all affected patients displayed predominant axial ataxia with variable AO and variable ID. Furthermore, the presence of dysmetria, gaze-evoked nystagmus, impaired optokinetic nystagmus and minipolymyoclonus was variable (Table 1). The index case had a predominant appendicular ataxia. None of the examined patients had microcephaly, dysmorphism, paroxysmal movements (chorea/ataxia) or pyramidal signs. Reflexes were normal in all examined patients except for III:1, who had hyporeflexia and polyneuropathy. Cognitive profiles were variable, ranging from low level (II:5, IV:1), border line level (III:2) to manifest ID (III:1); all the examined patients have attended special schools (Table 3). Patient IV:1 remained illiterate. The index case (Patient IV:1) had anger outbursts; she and her mother were diagnosed with ADHD.

**Patient I:1:** This woman was described as being affected by tremor and a gait disorder by her relatives. Age of onset for tremor was 23 years according to medical records. The tremor was slowly progressive; the patient developed unsteadiness during the course of disease. At age 81 the patient was evaluated at a different hospital due to tremor exacerbation; starting in her legs with spread to the rest of her body. The patient was able to ambulate with a walker. In addition, dysmetria, dysarthria and nystagmus were evident but her symptoms were interpreted as compatible with essential tremor. Rigidity was also documented; despite that description, there was not mentioning of either bradykinesia or orthostatic tremor. A year later, the patient lost her ability to walk. A brain CT scan displayed WMA but no cerebellar atrophy. The patient died at the age of 87 years.

**Patient II:2**: Next-of-kin perceived onset of a gait disorder at the age of 30 years with slow progression. His past medical history (PMH) consisted of falls and 3 fractures: at age 40 in the left foot, at age 41 in the left patella and at age 70 in the right femur; the latter motivated prosthetic surgery after which a bone density scan demonstrated osteoporosis and the patient was put on treatment with a bisphosphonate. He also suffered from DVT 3 times and pulmonary embolism, the patient harbors a heterozygous mutation in factor II and has been treated with warfarin ever since. The patient has attended a special school and has worked as a crane operator but retired at age 44 years, shortly after a car accident with subsequent whiplash symptomatology. Examination at age 65 revealed predominant axial ataxia, nystagmus, weak horizontal OKN, absent vertical OKN, moderate dysmetria, and minipolymyoclonus. In addition, dysarthria was mild and finger taps were slow. After that first visit, he started to use a walker more frequently. Screening with MoCA yielded 22 points, demonstrating deficits in visuospatial and attention tasks. The patient declined further psychometric testing, neuroimaging and neurophysiological exams.

**Patient II:5:** This man attended a special school. He has been able to write his name and numbers only but never learned how to read. His PMH consists of hypercholesterolemia, AO for a slowly progressive gait disorder was 18. At age 43 years the patient was evaluated at a different center for complaints of dizziness and unsteadiness. Physical examination at that point demonstrated ataxia, dysarthria and nystagmus. Aa brain MRI at this point was interpreted as normal. Examination at age 60 years demonstrated predominant axial ataxia, moderate dysmetria, nystagmus and weak horizontal and vertical OKN. At this age, his SARA score was 17 and MoCA yielded 18 points. In addition, minipolymyoclonus was found whereas reflexes were normal; his thenar muscles were mildly atrophic and an ENeG demonstrated severe carpal tunnel syndrome but the patient declined surgery. At age 61, the patient presented with multiple swollen joints in his hands, knees and feet. He was diagnosed with rheumatoid arthritis (RA) and treated with cortisone first and later with leflunomide and prophylaxis for osteoporosis. RA worsened his gait difficulties and increased the SARA score transiently (25 p. as worse). At age 63, his SARA score was 18.5 points. This patient remains illiterate; he had a total education of 9 years in a special school. The patient lives alone and despite his disabilities has worked part time as a cleaner and janitor. Psychometric testing at age 63 yielded an extremely low result in the brief cognitive status examination Mini Mental Test. The general intellectual ability according to the Ravens progressive matrices was in the lower normal range (IQ=92). A result considerably below average was found in tests assessing the following domains: visuospatial episodic memory (ROCFT/immediate and delayed recall, working memory (Digit span), spatial/visual construction (ROCFT copy), information processing speed (SDMT). He obtained a low average result in a test measuring verbal episodic memory/learning (RAVLT/learning) and an average result in long term verbal retrieval (RAVLT/long term verbal retention).

**Patient III:1** was diagnosed with type 1 diabetes mellitus (T1DM) at age 9; at age 19, the patient presented with delusions, auditory and visual hallucinations and carried out several self-harming acts and serious suicide attempts (e.g. injecting high insulin dosages). These symptoms were diagnosed as psychotic syndrome psychotic disorder not otherwise specified and emotionally unstable personality disorder. The patient meets the current DSM-5 criteria for both conditions. She has been treated with different neuroleptics ever since; depressive symptoms diagnosed later motivated treatment with mirtazapine. At age 25, she was hospitalized due to suspected self-induced hypoglycemic episode with obtundation. A few days after discharge she developed insidious tremor and unsteadiness that motivated evaluation. Dysmetria, dysarthria, nystagmus and broad-based gait were documented then; the initial interpretation was hypoglycemia-related movement disorder. However, her ataxia has been clearly progressive, motivating the use of a walker. Dysphagia appeared later on. Examination at age 43 revealed a marked axial ataxia, titubation, staccato speech, pronounced dysmetria, mild posturing and bradykinesia. Gabapentin was added to attenuate her tremor. This patient has obesity, two other affected have BMI >25 (III:2 and IV:1).

**Patient III:2**: Right after birth, cervical dystonia and tremor were noticed according to available medical charts. These features were exacerbated by fever and persisted during early childhood but it is unclear at what age the cervical dystonia resolved. Examination at age 2 revealed broad-based gait and head tilting. Nystagmus was documented two years later for the first time and the patient was diagnosed with familial cerebellar ataxia. A brain CT scan at age 2 years was normal. The patient avoided sport activities at school and has reported an inability to keep a straight line when walking for as long as she recalls. Delayed motor and language development were also documented; at age 5 years her speech was described as slurred. She was able to build complete and clear sentences at age 6.5. The patient attended special classes ever since. At age 24 years, the patient was treated for one depressive period. At age 33, the patient was evaluated and considered to meet the DSM-5 criteria for ADHD but pharmacological treatment was withheld due to a good functional level. Examination at this point demonstrated moderate axial ataxia, mild dysmetria, nystagmus, weak OKN in both vertical and horizontal directions and minipolymyoclonus. Her SARA score was 5.5 points only and MoCA at this age yielded 26 points. At age 39, her SARA score was 7 points. Difficulties with learning and attention were noticed early and the patient attended a special school. A psychometric evaluation at age 33 that included assessment with WAIS-IV revealed a general intellect clearly below average with a whole scale IQ = 71. A result beneath average was found in working memory (digit span), spatial/visuoconstructive test (Rey Complex Figure Test/copy) and in a test measuring visuospatial episodic memory (Rey Complex Figure Test/short- and long term retention). The patient works full-time as a personal assistant to patients with motor disabilities.

**Patient IV:1:** The index case in this family was first investigated at age 11 years for anger outburst and cognitive decline. Her previous diagnosis was congenital nystagmus and the family history was initially overseen. Tremor and learning difficulties were noticed at the age of 6 years. She was diagnosed with attention-deficit/hyperactivity disorder (ADHD) two years later (The patient meets the DSM-5 criteria for ADHD). Her past medical history consisted of hypothyroidism treated with levothyroxine. Initial treatment attempts with methylphenidate exacerbated her anger outburst and were discontinued; dexamphetamine induced tachycardia and was interrupted. Upon examination, both gaze-evoked and vertical nystagmus, postural and action tremor, mild dysmetria and impaired ability to do tandem gait were evident. In addition, mild tilting of the head, weak vertical OKN and minipolymyoclonus were found. She was put on propranolol for tremor with some alleviation. At age 9, a neuropsychological investigation was performed in order to determine the need for special education. The Wechsler Intelligence Scale for Children (WISC-IV) was administered. Her General Ability Index (GAI) according to WISC-IV was at low average in relation to her age group (IQ=72-82). In WISC-IV the verbal subtests were generally in a low average range while the performance tests overall yielded results in the average range. The performance in working memory tasks as well as speed was considerably below average. Her visuomotor coordination was also clearly below average. The conclusion of this investigation was that the patient had dyscalculia and dyspraxia. A second cognitive evaluation at age 13 yielded similar results as the evaluation 4 years before. The patient attended a special school from age 12. Neuroimaging at ages 11 and 17 demonstrated a cyst in the pineal gland, which in the context is an incidental finding. No other imaging abnormalities were found; in addition EEG and ENeG were normal. During follow-up her BMI has increased to 30.

- 1. **Cognitive features:**

Briefly, all the affected patients display variable degrees of ID (findings are summarized in table 3), requiring special education. The index case and Patient III:2 were scheduled to psychometric assessment at our center but chose not to attend these sessions.

- 1. **Neuroimaging**

Besides what is stated in the main manuscript and displayed in Figures 2-4, no significant abnormalities compared to normative data were identified in volumetric analyses^6^; however, there were trends towards large enlarged ventricles and low volumes of the thalami and corpus callosum (data not shown). 3D-SSP demonstrated a consistent pattern of hypometabolism in the prefrontal cortex (PFC), temporal cortex and cerebellum (Table 2).

- 1. **Genetics**

The ~600 kb deletion on chromosome 13q33 affects both *FGF14* and *ITGBL1* (Figure 5). Minimal region using Hg19 was determined by the coordinates chr13:102,108,769-102,707,510 = 13q33.1 *FGF14* is spliced into two isoforms *FGF14-1A* (27.7 kDa) and *FGF14-1B* (28.5 kDa) ^7^*.*

**References**

1. Paucar M, Bergendal Å, Gustavsson P, *et al*. Novel Features and Abnormal Pattern of Cerebral Glucose Metabolism in Spinocerebellar Ataxia 19. Cerebellum. 2018;17(4):465-76.
2. Fischl B. FreeSurfer. Neuroimage. 2012;62(2):774-81.
3. Wahlund LO, Barkhof F, Fazekas F, *et al*. A new rating scale for age-related white matter changes applicable to MRI and CT. Stroke. 2001;32(6):1318-22.
4. Wattjes MP, Henneman WJ, van der Flier WM, *et al*. Diagnostic imaging of patients in a memory clinic: comparison of MR imaging and 64-detector row CT. Radiology. 2009;253(1):174-83.
5. Alshammari MA, Alshammari TK, Laezza F. A Improved Methods for Fluorescence Microscopy Detection of Macromolecules at the Axon Initial Segment. Front Cell Neurosci. 2016;10:5. eCollection 2016.
6. Potvin O, Mouiha A, Dieumegarde L, *et al*. FreeSurfer Normative data Data Brief. 2016;9:732-736.
7. Miura S, Kosaka K, Fujioka R, *et al*. Spinocerebellar ataxia 27 with a novel nonsense variant (Lys177X) in FGF14. Eur J Med Genet. 2019;62(3):172-6.
